# Gray and White Matter Changes Associated with Psychophysical Functions Induced by Diabolo Training in Young Men

**DOI:** 10.3390/tomography8020070

**Published:** 2022-03-21

**Authors:** Ming-Chung Chou, Jui-Hsing Lin, Ming-Ting Wu

**Affiliations:** 1 Department of Medical Imaging and Radiological Sciences, Kaohsiung Medical University, Kaohsiung 80708, Taiwan; mcchou@kmu.edu.tw; 2 Department of Medical Research, Kaohsiung Medical University Hospital, Kaohsiung 80708, Taiwan; 3 Center for Big Data Research, Kaohsiung Medical University, Kaohsiung 80708, Taiwan; 4 Department of Physical Education, National Pingtung University, Pingtung 900391, Taiwan; rayshing@mail.nptu.edu.tw; 5 Department of Radiology, Kaohsiung Veterans General Hospital, Kaohsiung 813414, Taiwan; 6 Faculty of Medicine, School of Medicine, National Yang Ming Chiao Tung University, Taipei 11221, Taiwan; 7 Institute of Clinical Medicine, National Yang Ming Chiao Tung University, Taipei 11221, Taiwan

**Keywords:** brain plasticity, gray matter, white matter, diabolo training, psychophysical test

## Abstract

Learning a skill has been demonstrated to relate to neural plasticity in both animal and human brains. Performing diabolo consists of different tricks and may cause brain structural changes associated with psychophysical functions. Therefore, the purpose of this study was to investigate gray matter (GM) and white matter (WM) changes associated with psychophysical functions induced by diabolo training in healthy subjects. Fourteen healthy right-handed male subjects were enrolled to receive the diabolo training. Whole brain T1-weighted images and diffusion tensor imaging (DTI) data were acquired from all subjects on a 3.0 T magnetic resonance scanner before and after the training. Voxel-based morphometry (VBM), surface-based morphometry (SBM), and voxel-wise DTI analysis were carried out to detect the GM volume, cortical thickness, and WM diffusion changes using T1-weighted image and DTI data, respectively. In addition, two-arm coordination and mirror-drawing tests were performed to evaluate their psychophysical functions before and after 2, 4, 6 and 8 weeks of training. Analysis of variance was performed to understand whether the psychophysical functions changed over time after the training. The results showed that the psychophysical functions were significantly changed over time during the training. The VBM and SBM analyses revealed that the GM volume and cortical thickness were significantly increased in the brain areas associated with visual, motor, sensory, and spatial cognition functions. The voxel-wise DTI analysis further demonstrated that the mean diffusivity was significantly reduced in the genu of corpus callosum. Moreover, significant correlations were revealed between the increase rate of GM volume and the improvement rate of psychophysical functions in the left angular gyrus. The results suggest that the diabolo training may induce increased GM volume associated with improved psychophysical function in the brain region involved in spatial cognition and attention. Therefore, we conclude that the diabolo training may improve psychophysical function which might be reflected by the increased GM volume in the angular gyrus.

## 1. Introduction

Neural plasticity has been widely accepted to explain why humans are able to adapt a changing environment or to learn a novel skill, and it may be associated with brain structural and functional changes in task-related brain regions. Mouse brain studies have shown that learning (including traversing rope ladder, high step, wide boards, chain, weather stripping, and seesaw) could lead to synaptogenesis and glial hypertrophy, but an increase in motor activity only results in angiogenesis [1,2]. In addition, electrical activity could regulate the myelination of axons in white matter (WM) [3,4]. These evidences suggest that both gray matter (GM) and WM changes could be induced by learning or by external stimulus in animal brains.

In human studies, previous studies have performed magnetic resonance high-resolution T1 voxel-based morphometry (VBM) analysis and demonstrated that young adults, who were trained to perform three-ball cascade juggling, were found to have increased cortical GM volume and cortical thickness [5,6,7]. Some studies demonstrated that GM volume is associated with a rate of skill learning after long-term training intervention [8], and that elderly persons had less proficiency to learn three-ball cascade juggling with a less increased GM volume than 20-year-old adolescents [9]. In addition to GM changes, one previous study utilized voxel-wise diffusion tensor imaging (DTI) analysis and showed that learning to juggle could lead to increased fractional anisotropy (FA) values in the WM [10]. Therefore, high-resolution T1-VBM and voxel-wise DTI analyses are helpful to detect GM and WM changes induced by learning juggling in the human brain, respectively.

In Asian countries, performing diabolo (double-cone bobbin), a traditional sport, is more popular than doing multi-ball cascade juggling. Performing diabolo is different from cascade juggling because one needs to use both hands to control a diabolo through two sticks connected by a rope and it consists of many tricks, including acceleration, elevator (ladybug), toss, knot (magic knot), trapeze, spidernet, grind, and orbit, which are involved in visual, spatial cognition, motor, and somatosensory functions of the brain. It was demonstrated that the diabolo exercise is beneficial to body health and may have a positive effect on emotion and sleep in adults [11]. In multi-ball cascade juggling, previous studies have shown that the GM volume was increased in the right inferior occipital, left middle occipital, left precuneus, right superior orbital frontal, left superior frontal, bilateral middle temporal, and bilateral anterior cingulate [5,6] as well as WM changes in the intraparietal sulcus [10]. However, it remains unclear how the brain GM and WM were changed after the diabolo exercise.

In addition, learning a sport may enhance one’s motor or psychophysical functions which could be assessed by two-arm coordination [12] and mirror-drawing test [13,14,15]. Both tests were able to assess the performance of motor learning and physical activities because they involved the gaze, motor, and visual systems. As psychophysical functions and corresponding brain changes may be enhanced by diabolo training, we hypothesized that the diabolo training may improve psychophysical functions reflected by the changes of brain structure in specific brain regions. The purpose of this study was to investigate the relationship between the changes of GM volume, cortical thickness, WM diffusion, and psychophysical functions after diabolo training, using VBM, surface-based morphometry (SBM), and voxel-wise DTI analyses, respectively.

## 2. Materials and Methods

This prospective study was approved by the local institutional review board (protocol number: 970067R). Fourteen male subjects (age = 22.6 ± 2.6 years) were successfully enrolled in this study. The inclusion criteria were as follows: (1) age ≥ 20 years old; (2) right handedness; (3) Asian race; (4) no previous experience of diabolo; and (5) no history of neurological, psychological, and physical disorders. By providing their written informed consents, all subjects were arranged to have an initial magnetic resonance imaging (MRI) scan to examine whether they had pre-existing brain lesions by a senior radiologist (M.T.W.), and if not, the acquired MR images were used to measure their GM volume, cortical thickness, and WM diffusion. Subsequently, all subjects as a group began to receive 8-week-long (2 days/week and 40 min/day) diabolo training with several fundamental tricks, including acceleration, elevator (ladybug), toss, knot (magic knot), trapeze, spidernet, grind, and orbit. The diabolo training was supervised by a professional coach, and the progress of the diabolo skills were recorded during the training period. All subjects consistently attended the training course without leave. In the meantime, two kinds of psychophysical tests (two-arm coordination and mirror drawing) were performed on all subjects before and after 2, 4, 6, and 8 weeks of training to evaluate the longitudinal changes in psychophysical functions. In the present study, all subjects were able to correctly perform those fundamental tricks without making a mistake or dropping the diabolo since the sixth week of training. At the end of diabolo training, a second MRI scan was performed on all subjects to measure their GM volume, cortical thickness, and WM diffusion for comparisons.

### 2.1. Psychophysical Tests

**Figure 1:**
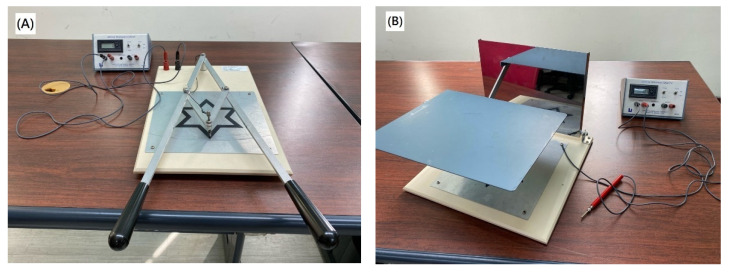
The instruments used for two-arm coordination (**A**) and mirror-drawing (**B**) tests.

In this study, two kinds of psychophysical tests were performed on all subjects before and after 2, 4, 6, and 8 weeks of the diabolo training. One is the two-arm coordination test, and the other is a mirror drawing test. These two tests were performed with two kinds of instruments containing a six-pointed star pattern (Lafayette Instrument, Lafayette, IN, USA). In the two-arm coordination test, each subject was asked to hold two handles to control a stylus by tracing a six-pointed star pattern on the testing board in a clockwise direction as quickly and as accurately as possible, as shown in Figure 1A. A timer was operated manually to record the total elapsed time (T) in seconds that subjects complete the tracing, and a counter automatically records the total number of errors (N) that the stylus leaves the star pattern during the test. As both the T and N reflect the performance of the motor coordination, the present study calculated the combined score, defined as T/2 + N [16], to evaluate the subject’s overall function of motor coordination. 

In the mirror-drawing test, all subjects were asked to hold a stylus using their right hands by tracing a mirror-reflected (i.e., reversed) six-pointed star pattern on a testing board in a clockwise direction as quickly and as accurately as possible, as shown in Figure 1B. Similarly, a timer was used to manually record the total elapsed time, and a counter was used to automatically record the total number of errors that the stylus leaves the star pattern during the test. The score, also defined as T/2 + N [16], was calculated to evaluate the subject’s overall psychophysical function.

### 2.2. MRI Acquisition

MRI scans were performed on all subjects using a 3.0 T MR scanner (Magnetom, Siemens, Erlangen, Germany) with a 12-channel phased-array head coil. After tri-planar scan for localization, whole-brain high-resolution T1-weighted imaging data (TR/TE/TI = 2530/3.5/1100 ms, flip angle = 7 degree, field-of-view = 256 × 256 × 176 mm, matrix size = 256 × 256 × 176, bandwidth = 190 Hz/pixel, scan time = 10 min) and DTI data (TR/TE = 7500/72 ms, field-of-view = 256 × 256 mm, matrix size = 128 × 128, slice thickness = 2 mm, b-value = 1000 s/mm^2^, signal-to-noise ratio of b0 image = 20, number of direction = 12, number of excitation = 6, acceleration factor = 4, scan time = 11 min) were acquired using 3D magnetization prepared rapid gradient echo and spin-echo echo-planar diffusion-weighted pulse sequences, respectively. The acquisition was performed with identical imaging parameters before and after the diabolo training.

### 2.3. Voxel-Based Morphometry

**Figure 2:**
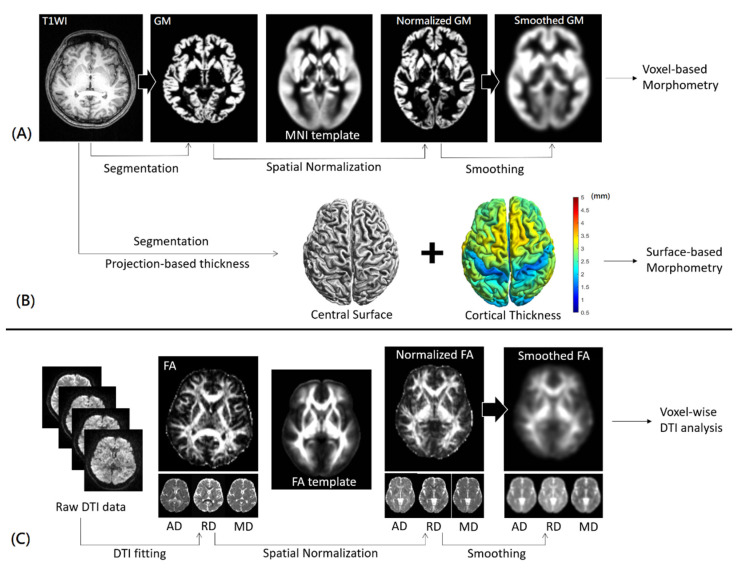
The schematic diagram for voxel-based morphometry (**A**), surface-based morphometry (**B**), and voxel-wise DTI (**C**) analyses. T1WI = T1-weighted image. GM = gray matter; MNI = Montreal Neurological Institute; DTI = diffusion tensor imaging; AD = axial diffusivity; RD = radial diffusivity; MD = mean diffusivity; FA = fractional anisotropy.

The high-resolution T1-weighted imaging and DTI data were transferred to a standalone workstation and were post-processed using Computational Analysis Tool version 12 (CAT12, University of Jena, Jena, Germany) (http://www.neuro.uni-jena.de/cat/, accessed on 20 March 2022), FMRIB Software Library (FSL, Oxford University, Oxford, UK) (https://fsl.fmrib.ox.ac.uk/fsl/fslwiki/, accessed on 22 March 2022), and Statistical Parametric Mapping version 12 (SPM12, University College London, London, UK) (https://www.fil.ion.ucl.ac.uk/spm/software/spm12/, accessed on 19 March 2022). In VBM analysis, the CAT12 toolbox was utilized because it was previously demonstrated to provide better results than VBM8 in detecting GM changes in patients with temporal lobe epilepsy [17]. The CAT12-VBM consists of several pre-processing steps, including field bias modulation, tissue segmentation, diffeomorphic anatomical registration through exponentiated lie algebra-based spatial normalization [18], and spatial smoothing. In this study, the VBM analysis was carried out with default parameters (bias correction = “rough”, strength of local adaptive segmentation = “medium”, voxel size for normalized images = 1.5 mm, internal resampling = fixed 1.0 mm, smooth kernel full width at half maximum (FWHM) = 8 mm) and East Asian brain template, and the normalized GM images were statistically compared on a voxel-by-voxel basis. The schematic diagram for VBM analysis is shown in Figure 2A.

### 2.4. Surface-Based Morphometry

In SBM analysis, this study also utilized the CAT12 toolbox to estimate the cortical thickness and central surface using the projection-based thickness method [19]. In this study, the SBM analysis was carried out with default parameters (bias correction= “rough”, strength of local adaptive segmentation = “medium”, voxel size for normalized images = 1.5 mm, internal resampling = fixed 1.0 mm, resample size = 32k mesh, smooth kernel FWHM = 15 mm) and the East Asian brain template, and the central surface and cortical thickness were statistically compared on a vertex-by-vertex basis. The schematic diagram for SBM analysis is shown in Figure 2B.

### 2.5. Voxel-Based DTI Analysis

In voxel-based DTI analysis, motion correction was first performed using rigid-body registration, from which the rotation matrix was used to compensate for diffusion directions [20]. Then, affine registration was performed to minimize the eddy-current distortions in diffusion data using FLIRT (FMRIB’s Linear Image Registration Tool, Oxford University, Oxford, UK), followed by a brain extraction using Brain Extraction Tool (BET) tool to remove non-brain signals. Subsequently, a DTI model was fitted on a voxel-by-voxel basis using the DTIFIT tool to obtain axial (AD), radial (RD), mean diffusivity (MD), and fractional anisotropy (FA). Moreover, the FA maps were spatially normalized to an international consortium for a brain mapping-FA template using both linear affine and non-linear diffeomorphic demon registrations [21], which was demonstrated to outperform several non-linear registration techniques [22]. The displacement maps were utilized to spatially normalize the corresponding AD, RD, and MD map. Finally, the normalized maps were smoothed with a kernel of FWHM = 8 mm for voxel-wise comparisons. The schematic diagram for voxel-wise DTI analysis is shown in Figure 2C.

### 2.6. Statistical Analysis

For psychophysical scores, one-way repeated analysis of variance (ANOVA) was performed to test whether the scores were significantly changed over time during the diabolo training, and the changes were considered significant if corrected *p* < 0.05 with Bonferroni correction. The brain structural changes induced by diabolo training were detected using a voxel-wise paired *t*-test of SPM12 toolbox to understand the global changes in GM volume and DTI indices in the subjects after 8 weeks of diabolo training, and the changes were considered significant if a cluster-level family-wise error (FWE) corrected *p* < 0.05 (uncorrected *p* < 0.001 and cluster > 100 voxels). For cortical thickness, vertex-based analysis was performed using the CAT12 toolbox to reveal the changes in cortical thickness in the subjects with diabolo training, and the changes were considered significant if the cluster-level FWE corrected *p* < 0.05 (uncorrected *p* < 0.01 and cluster > 100 vertices). In significant regions, a Pearson’s correlation coefficient was calculated to understand the associations between change rates in GM volume, cortical thickness, DTI indices, and psychophysical scores after the diabolo training, as well as the associations between those changes and subjects’ age. The correlation analysis was performed using IBM SPSS Statistics for Windows (Version 20.0, IBM Corporation, New York, NY, USA), and the results were considered significant if uncorrected *p* < 0.05.

## 3. Results

**Figure 3:**
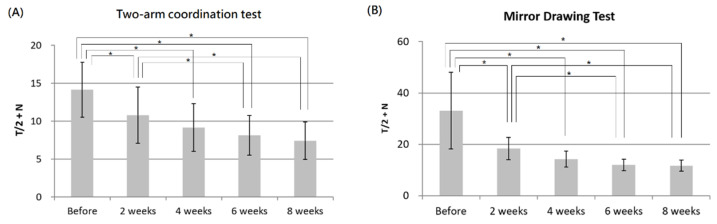
The results of the two-arm coordination (**A**) and mirror-drawing (**B**) tests measured in the subjects before and after 2, 4, 6, and 8 weeks of the diabolo training. In ANOVA analysis, the F-scores are 10.81 and 22.72 for the two-arm coordination and mirror drawing tests, respectively. Asterisks (*) indicate *p* < 0.05 with Bonferroni correction.

In psychophysical tests, ANOVA analysis demonstrated significant changes in both two-arm coordination and mirror-drawing scores over time during 8 weeks of diabolo training. The post hoc analysis further revealed that both two-arm coordination and mirror-drawing scores were significantly changed after 2, 4, 6 and 8 weeks of training. In addition, significant differences were also noted between 2 and 6 weeks as well as between 2 and 8 weeks of training, as shown in Figure 3.

**Figure 4:**
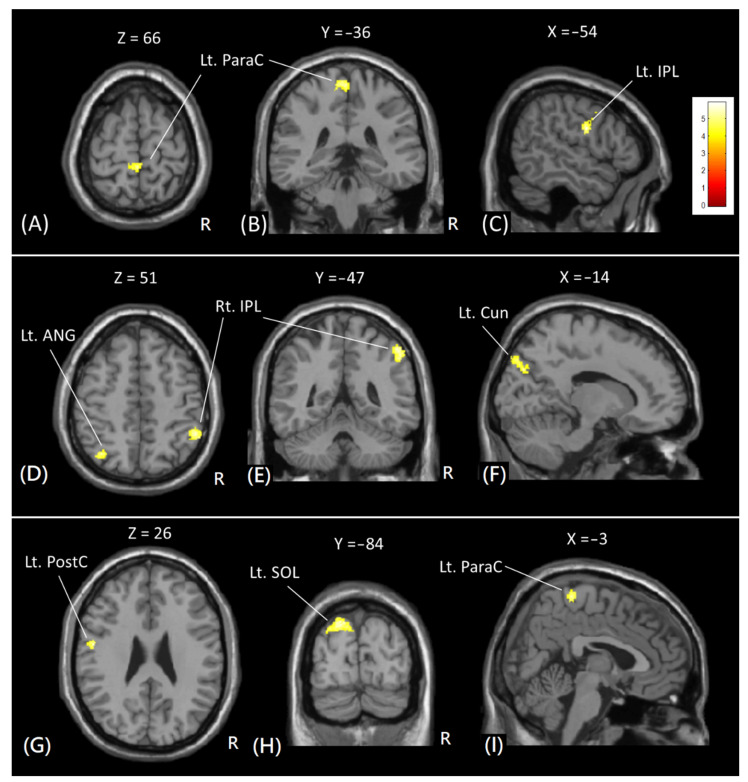
The voxel-based comparison of the GM volume in subjects before and after diabolo training. Red-yellow colors indicate increased GM volumes after training. Images are shown in axial (**A**,**D**,**G**), coronal (**B**,**E**,**H**), and sagittal (**C**,**F**,**I**) views. The color bar in the upper right corner indicates the T-value. Lt = left; Rt = right; ParaC = paracentral gyrus; PostC = postcentral gyrus; IPL = inferior parietal lobule; ANG = angular gyrus; Cun = Cuneus; SOL = superior occipital lobule.

In VBM analysis, significant increases in GM volume were found in the right inferior parietal lobule, left superior occipital lobule, left cuneus, left middle occipital gyrus, left postcentral gyrus, left angular gyrus, and left paracentral lobule after 8 weeks of diabolic training, as shown in Figure 4. However, no significant decrease in GM volume was noted. Table 1 lists the Brodmann area (BA) and Montreal Neurological Institute (MNI) coordinate of significant clusters with increased GM volume with cluster-level FWE-corrected *p* < 0.05.

**Figure 5:**
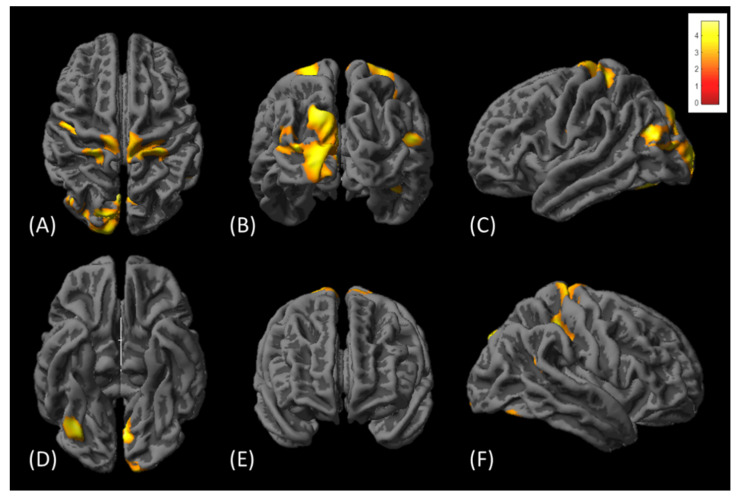
The comparison of cortical thicknesses before and after diabolo training. Red-yellow colors superimposed on the brain surface indicate significant increase in cortical thickness after the training. The brains are viewed from (**A**) top, (**B**) posterior, (**C**) left lateral, (**D**) bottom, (**E**) anterior, and (**F**) right lateral sides, respectively. The color bar in the upper right corner indicates the T-value.

In surface-based cortical thickness analysis, the cortical thicknesses were significantly increased in multiple regions, involving bilateral precentral, bilateral postcentral, bilateral cuneus, bilateral occipital, left superior parietal, left fusiform, right lingual, and right supramarginal gyrus, as shown in Figure 5. However, no significant decrease in cortical thickness was detected in those subjects. Table 2 lists cortical regions with significant increases in cortical thickness with cluster-level FWE-corrected *p* < 0.05.

**Figure 6:**
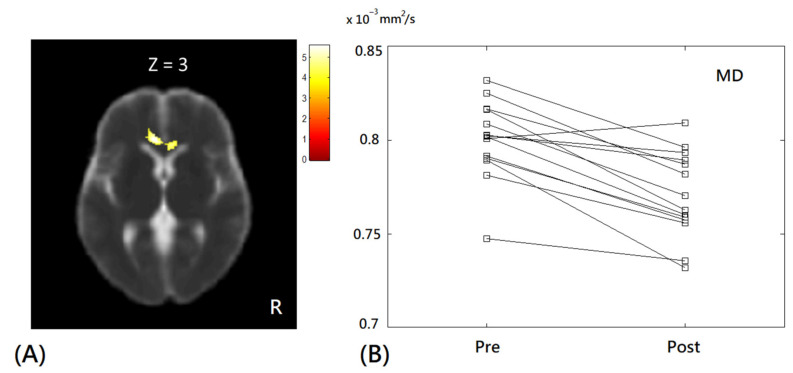
The voxel-based comparison of MD values in subjects before and after diabolo training. Red-yellow colors superimposed on the image indicate significantly decreased MD values after the training. (**A**) Axial MD map with a significant cluster in the corpus callosum. The color bar in the upper right corner indicates the T-value. R = right. (**B**) Paired scatter plot of MD values before (pre) and after (post) diabolo training. MD = mean diffusivity.

In voxel-based DTI analysis, significant decreases in MD values were only observed in the genu of corpus callosum after diabolo training, as shown in Figure 6; however, no significant change of AD, RD, and FA value was found in the subjects.

**Figure 7:**
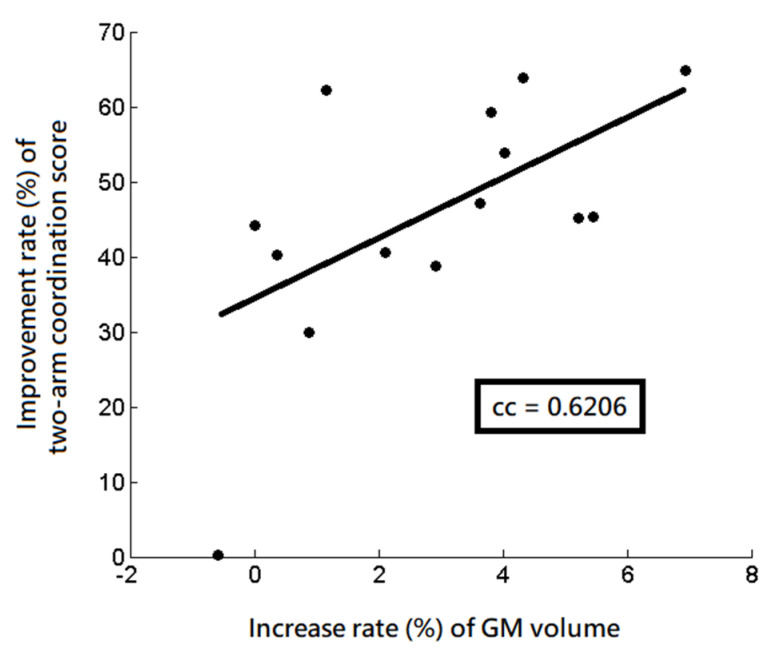
The significant correlation between the increase rate of the GM volume and the improvement rate of the two-arm coordination in the left angular gyrus after diabolo training. cc = correlation coefficient.

In those significant regions detected by VBM, SBM, and DTI analyses, the following correlation analysis revealed a significantly positive correlation between the increase rate (%) of the GM volume and the improvement rate (%) of the two-arm coordination function (r = 0.6206, *p* = 0.018) in the left angular gyrus, as shown in Figure 7. However, no significant correlation was noted between the changes in MD values, cortical thickness, psychophysical scores, and age.

## 4. Discussion

To the best of our knowledge, this is the first MRI study to investigate both GM and WM changes associated with psychophysical functions induced by diabolo training. Our results demonstrated that, after 8 weeks of diabolo training, the subjects exhibited significant increases in psychophysical functions (both two-arm coordination and mirror drawing), GM volume, and cortical thickness in multiple brain regions. The significantly increased GM volume and cortical thickness were located in regions related to functions of motor (precentral and paracentral gyrus), sensory (postcentral, paracentral, inferior parietal, superior parietal, and supramarginal), visual (superior occipital, middle occipital, superior parietal, cuneus, angular, and lingual gyrus), spatial cognition and attention (superior parietal, angular, fusiform). These findings suggest that learning diabolo might enhance visual, somatosensory, motor, and spatial cognition functions in human brain. 

The increase in GM volume in the brain may likely be due to the enlargement of cell size, the genesis of neural or glial cells, or changes in the blood flow or extra-cellular fluid [23]. Previous animal studies suggest that learning may lead to synaptogenesis and glial hypertrophy, but an increase in motor activity only results in angiogenesis [1,2]. Previous human studies also demonstrated that healthy adults who were trained to do three-ball cascade juggling had increased cortical GM volume and thickness [5,6,7]. In the present study, performing diabolo was found to lead to significant increases in both GM volume and cortical thickness, which could among other causes, reflect changes in synaptogenesis and/or glial hypertrophy [1,2]. 

We found a significant positive correlation between increase rate of GM volume and the improvement rate of two-arm coordination scores in the left angular gyrus. The angular gyrus has been known to be involved in spatial cognition and attention in many tasks [24], suggesting that the increased GM volumes were associated with improved motor coordination in the subjects receiving 8 weeks of diabolo training. 

In addition to GM changes, previous animal studies have suggested that electrical activity could regulate the myelination of axons [3,4] which may be reflected by the alteration of diffusion properties, either MD or FA, in the tissue. A previous human study demonstrated that learning to juggle could lead to increased FA values in the WM region (intraparietal sulcus) [10]. In contrast we observed a significant decrease in MD values solely in the genu of corpus callosum, suggesting that learning to diabolo could lead to the modulation of myelination. The reduced diffusivity may indicate decreased extra-cellular space in the WM tissues, likely due to increased axonal density or glial hypertrophy after diabolo training. As the genu of corpus callosum consists of frontal inter-hemispheric fiber connections, the MD changes in the genu of corpus callosum may indicate that learning to diabolo may also alter cognitive functions [25]. Further investigation is worthy to understand the brain changes induced by diabolo training beyond the psychophysical tests. 

Diabolo training induced the expansions of GM volume and cortical thickness in areas that were partially consistent with 3-ball cascade juggling. In the juggling training, previous studies showed that the training induced the increases in GM volume in the right inferior occipital (BA18), left middle occipital (BA18), inferior parietal lobule (BA40), right superior orbital frontal (BA10), left superior frontal (BA10), bilateral middle temporal (BA21), bilateral anterior cingulate (BA24), and bilateral intra-parietal sulcus (BA7) [5,6]. Consistently, the present study demonstrated that diabolo training induced the increases in GM volume in the right inferior parietal lobule (BA40) and left cuneus (BA18), as well as induced the increases in cortical thickness in the left cuneus (BA18), right supramarginal gyrus (BA40), and left angular gyrus (BA7). These consistent findings suggest that both diabolo and juggling training are helpful for stimulating structural plasticity in the visual areas, which were likely associated with the common learning process of the juggling and diabolo trainings. 

Differently, the diabolo training did not induce brain changes in the superior frontal gyrus (BA10) and middle temporal gyrus (BA21) which were detected in previous juggling studies [5,6]. As both areas are responsible for cognition and distance contemplation, the inconsistency suggests that the juggling training may involve more brain functions in cognition and distance contemplation than the diabolo training. In addition, the present study demonstrated that the diabolo training additionally induced expansions of GM volume in the visual, motor, spatial cognition and attention areas (left superior occipital lobule (BA19), left middle occipital gyrus (BA19), left postcentral gyrus (BA43), and left paracentral lobule (BA4)), as well as the thickening of cortical thickness in the visual and motor areas (left middle occipital gyrus (BA19) and superior occipital gyrus (BA19)). These additional areas may suggest that the diabolo training might be a more demanding task that involved more cortical areas with motor, sensory, spatial cognition, and attention functions than the juggling training. 

Moreover, the inconsistent results may also be attributable to the difference of demographic characteristics, such as sex distribution and handedness, in subjects between our (14 right-handed male subjects) and previous (11 male and 9 female subjects [5]; 21 female and 3 male subjects [6]) studies. It has been shown that sex and handedness are associated with hemispheric asymmetry or brain lateralization [26]. Although the present study showed that the brain structures were changed in both hemispheres, there was a strong left lateralization of brain changes after the diabolo training. In general, playing diabolo requires both arms to do the tricks, but the motion of diabolo is mainly controlled by the dominant arm in most tricks. For example, in the acceleration trick, one needs to use the dominant arm to accelerate the diabolo by tossing it back and forth. Therefore, in the present study the brain changes in both hemispheres were likely due to the fact that diabolo requires both arms to do the tricks. However, the strong left lateralization of brain changes in the right-handed subjects may speculate that the lateralization towards the dominant hemisphere was likely attributable to the nature of performing diabolo, where the diabolo is mainly controlled by the dominant arm. 

There are some limitations that warrant discussion. First, a small sample size may not provide strong enough statistical power, and the correlation is very likely to be a false positive. Second, this study did not enroll control subjects, so the longitudinal changes in brain structure and psychophysical function without diabolo training cannot be assessed. The practice effect of multiple psychophysical tests has not been sufficiently confirmed. Third, this study only enrolled male subjects, so the findings were unable to reflect the brain changes in female subjects. Finally, we did not investigate the sustainability of the brain changes. A previous study demonstrated that the structural changes would decrease after ceasing to learn to juggle for a period longer than 2 weeks [5,6], so additional follow-up MRI scans with a “wash-out” period after the 8-week training may be helpful for this issue.

## 5. Conclusions

This study performed VBM, SBM, and voxel-wise DTI analysis to understand the changes in GM volume, cortical thickness, and WM diffusion in association with psychophysical functions in healthy subjects after diabolo training. It was noted that the increase rate of the GM volume was correlated with the improvement rate of two-arm coordination scores in the angular gyrus. We concluded that learning to diabolo may improve the psychophysical functions that might be reflected by the expansions of GM volume in the region involved in spatial cognition and attention functions.

## Figures and Tables

**Table 1:** The Brodmann area (BA) and Montreal Neurological Institute (MNI) coordinates of significant regions with increased GM volume after diabolo training.

Region	Brodmann Area	MNI Coordinate			Cluster Size (mm^3^)	Z Score of Peak-Level Difference
		X	Y	Z		
Rt. Inferior parietal lobule	40	51	−47	50	1110	3.98
Lt. cuneus	18	−14	−78	32	1789	3.78
Lt. Superior occipital lobule	19	−21	−84	44	1789	3.98
Lt. Middle occipital gyrus	19	−32	−84	36	1789	3.72
Lt. postcentral gyrus	43	−54	−5	26	567	3.89
Lt. angular gyrus	7	−40	−66	51	614	3.79
Lt. paracentral lobule	4	−3	−36	66	540	3.74

**Table 2:** The brain regions with a significant increase in cortical thickness in subjects after diabolo training.

Region	Brodmann Area	Cortical Thickness (mm)		FWE-Corrected *p* Value
		Before	After	
Lt. Cuneus	18	1.92 ± 0.13	1.99 ± 0.10	0.024
Lt. Middle occipital gyrus	19	2.66 ± 0.09	2.71 ± 0.09	0.046
Lt. Superior occipital gyrus	19	2.29 ± 0.14	2.36 ± 0.11	0.012

## Data Availability

The data are not publicly available due to the nature of this research, participants of this study did not agree for their data to be shared publicly.
